# Structural and biochemical characterization of the 3′-5′ tRNA splicing ligases

**DOI:** 10.1016/j.jbc.2025.108506

**Published:** 2025-04-10

**Authors:** Sebastian Chamera, Weronika Zajko, Mariusz Czarnocki-Cieciura, Marcin Jaciuk, Łukasz Koziej, Jakub Nowak, Krzysztof Wycisk, Małgorzata Sroka, Andrzej Chramiec-Głąbik, Mirosław Śmietański, Filip Gołębiowski, Marcin Warmiński, Jacek Jemielity, Sebastian Glatt, Marcin Nowotny

**Affiliations:** 1 Laboratory of Protein Structure, International Institute of Molecular and Cell Biology, Warsaw, Poland; 2 Malopolska Centre of Biotechnology (MCB), Jagiellonian University, Krakow, Poland; 3 Explorna Therapeutics sp. z o.o., Warsaw, Poland; 4 Division of Biophysics, Institute of Experimental Physics, Faculty of Physics, University of Warsaw, Warsaw, Poland; 5 Centre of New Technologies, University of Warsaw, Warsaw, Poland; 6 Department for Biological Sciences and Pathobiology, University of Veterinary Medicine Vienna, Vienna, Austria

**Keywords:** pre-tRNA splicing, protein complex, protein–nucleic acid interaction, protein structure, RNA-binding protein, RNA splicing, RTCB, structural biology, tRNA, tRNA ligase complex

## Abstract

In archaea and metazoa, tRNA exons are ligated by the RNA ligases RtcB and RTCB, respectively. The metazoan RTCB forms a stable complex with four additional subunits, DDX1, FAM98B, CGI99, and ASHWIN. The role and assembly of these four components remain elusive. Furthermore, we lack structural information of how RNA substrates are recognized by 3′-5′ tRNA ligases. Here, we use thiol-based chemical crosslinking to confirm the involvement of specific residues of RtcB in RNA binding, and we present a cryo-EM structure of the purified five-subunit *Danio rerio* tRNA ligase complex. The structure implies that the DDX1 helicase module is mobile and can modulate the activity of RTCB. Taken together, the presented results enhance our mechanistic understanding of RNA binding by 3′-5′ tRNA splicing ligases and architecture of the metazoan tRNA ligase complex.

Some archaeal and eukaryotic tRNAs are expressed as pre-tRNAs, which must undergo enzymatic splicing during maturation. Splicing occurs in two steps, namely endoribonucleolytic cleavage and ligation (1, 2). First, a tRNA endonuclease cleaves the pre-tRNA molecule at the two exon–intron boundaries. This results in three products, two tRNA exon halves with a 2′,3′-cyclic phosphate at the end of the 5′-exon and a 5′-hydroxyl at the end of the 3′-exon, and the excised linear intron (3, 4, 5, 6). The following ligation step of the two exons can occur *via* two different pathways, which differ in the origin of the phosphate that links the exon ends: (i) 5′-3′ phosphate ligation, also termed "healing and sealing" and (ii) 3′-5′ phosphate ligation, also known as "direct ligation" (3, 7, 8, 9, 10).

In archaea and metazoa, ligation of tRNA exons is almost exclusively achieved by the direct ligation mechanism that is very similar for metazoan and archaeal enzymes. The process starts by the excision of tRNA intron by a specific endonuclease EndA in archaea and TSEN complex in eukaryotes (11). The substrate is then handed over to the ligase, but the exact mechanism of this handover is not known. The RNA ligase RtcB catalyzes the GTP- and Mn^2+^-dependent ligation of the terminal 2′,3′-cyclic phosphate to the 5′-hydroxyl terminus (Fig. 1*A*) (12, 13, 14, 15, 16, 17, 18, 19, 20). In addition to its ligase activity, RtcB possesses a 2′,3′-cyclic phosphodiesterase activity that is used to first hydrolyze the 2′,3′-cyclic phosphate and convert it into a 3′-phosphate in one of tRNA exons (13, 17, 18). Subsequently, the ligation reaction proceeds through three nucleotidyl-transfer steps: (i) protein guanylation of RtcB, where GTP (pppG) is converted to GMP (pG) and the remaining phosphate forms a covalent bond with an active site histidine, (ii) transfer of the pG moiety to the phosphorylated 3′-end of the first tRNA exon to form an RNAppG intermediate, and (iii) the attack of the 5′-OH from the second tRNA exon on the activated 3′-phosphate to form a 3′,5′-phosphodiester bond and release of GMP (12, 13, 16, 17, 18, 19, 21, 22). In addition, to reach a high efficiency of activity, RtcB requires a protein cofactor—Archease (23, 24, 25). Archease reaches into the active site of RTCB and coordinates GTP and metal ions, promoting the formation of an RTCB–GMP intermediate (26). The *archaease* gene tends to be located adjacent to genes encoding proteins involved in DNA or RNA processing (23, 24, 25, 27).

**Figure 1:**
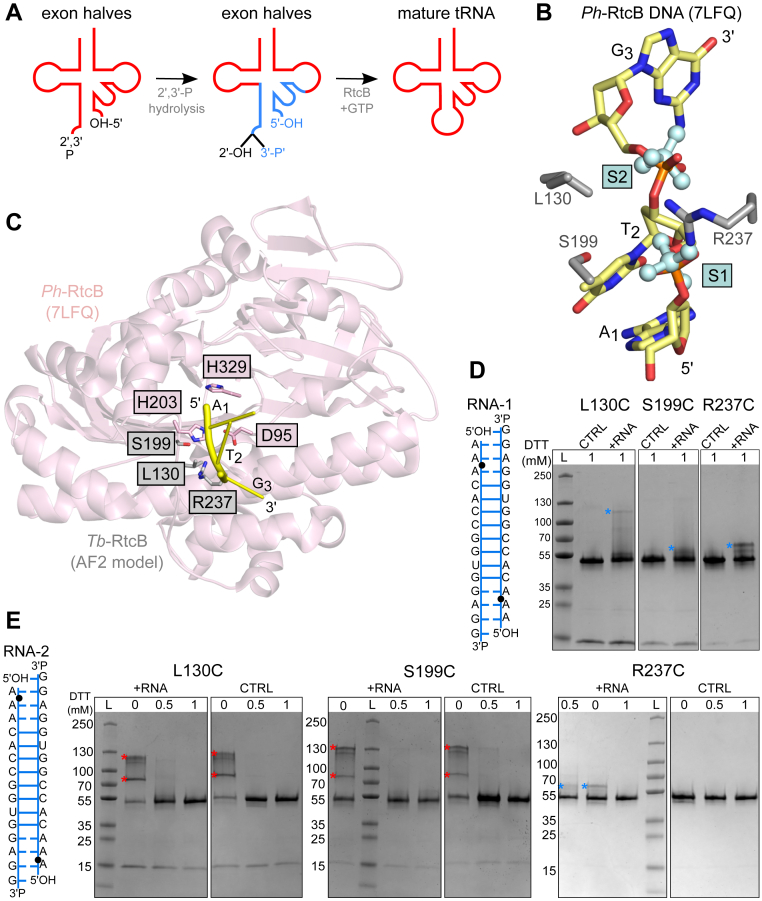
**Chemical crosslinking of *Tb*-RtcB and RNA.***A*, schematic representation of the ligation reaction performed by RtcB. *Blue part* corresponds to the fragment that was used for the design of RNA substrates for chemical crosslinking. *B*, structural analysis. AlphaFold 2 model of *Tb*-RtcB, structure of *Ph*-RtcB (PDB ID: 4ISJ), and structure of *Ph*-RtcB–DNA complex (PDB ID: 7LFQ) were superimposed. The panel shows sulfate (S1, S2) ions from 4ISJ structure (*cyan*), amino acid residues from the *Tb*-RtcB AlphaFold 2 model predicted to interact with RNA (shown as *gray sticks*), and DNA from *Ph*-RtcB–DNA complex structure (shown in *yellow*). *C*, same superposition as in (*B*) but with *Ph*-RtcB–DNA complex structure (PDB ID: 7LFQ) shown in *pink cartoon* for protein and DNA in *yellow*. Selected residues of AlphaFold 2 model of *Tb*-RtcB are shown as *gray sticks*. Active site residues of *Ph*-RtcB are shown as *pink sticks*. *D* and *E*, SDS-PAGE analysis of results of the cross-linking reactions of the *Tb*-RtcB mutants and the cystamine-modified RNA substrates. A schematic representation of the substrates is shown on the *left side* of the gels. *Black dot* represents the cystamine modification. In all cases, the *blue asterisks* mark specific crosslinks, whereas the *red asterisks* mark unspecific or protein–protein crosslinks. CTRL stands for reactions with protein only. L, molecular weight ladder; PDB, Protein Data Bank; *Ph*-RtcB, *Pyrococcus horikoshii* RtcB; *Tb*-RtcB, RtcB from archaeon *Thermococcus barophilus*.

The human tRNA ligase RTCB (an ortholog of the archaeal RtcB) forms a stable and active tRNA ligase complex (tRNA-LC), with four additional subunits: DDX1, FAM98B, CGI99, and ASHWIN (ASW) (12). However, the precise functional role of each of these four components is still unclear. The possible functions involve the localization of the complex in the appropriate cellular compartments, stabilization of the catalytic subunit, or the recruitment of specific substrates (12). DDX1 is a member of the DEAD-box family of putative RNA helicases and contains an SPRY domain. DDX1 has been implicated in functions, such as mRNA processing, DNA double-strand break detection, and 3′-5′ RNA unwinding activity. Binding of DDX1 to RTCB suggests a direct involvement in tRNA splicing, but its role in this process remains elusive (3, 28, 29, 30, 31, 32). Beyond the observation that CGI99 binds to a mature hepatitis C virus core protein to modulate the infection process (33, 34, 35), no specific function has been attributed to this protein. Likewise, the identification of FAM98B as a component of the human tRNA-LC constitutes the first report concerning its potential cellular function. Strikingly, the RNAi-mediated depletion of FAM98B has almost no impact on RNA ligase activity in HeLa cell extracts (3, 12). ASW was first characterized in a genetic screen performed in *Xenopus laevis* to identify genes that are differentially expressed during early neural specification (36).

In addition to tRNA ligation, RTCB also ligates *xbp1* mRNA, which has a function in the unfolded protein response—stress-signaling pathway that is activated in response to the accumulation of unfolded proteins in the endoplasmic reticulum (37). Moreover, archaeal and metazoan introns can be circularized into “tricRNAs”/tRNA intron circles by RTCB (38, 39, 40), and it is unknown how the enzyme targets these substrates in a timely manner.

Despite the large amount of structural data available for archaeal RtcBs, no structures have been reported that visualize the interaction between RtcB and an RNA substrate. At the moment, it is even unclear which amino acid residues of the ligase are involved in RNA binding. Moreover, the exact architecture of the metazoan tRNA-LC is unknown. So, despite many important roles of RtcB and RTCB complexes, their understanding is far from complete.

Here, we use a thiol-based site-specific chemical cross-linking approach to identify residues that are involved in RNA binding by archaeal RtcB. Furthermore, we reveal the molecular architecture of the tRNA-LC from *Danio rerio* by determining its single-particle cryo-EM structure.

## Results

### Molecular insight into RtcB–RNA interaction

Several structures of RtcB orthologs have been published (19, 20, 26, 41, 42, 43). However, we currently lack structural information on the RtcB enzyme in complex with its RNA substrates, and the exact RNA-binding region is not known. To address this, we purified RtcB from archaeon *Thermococcus barophilus* (*Tb*-RtcB and the catalytically inactive *Tb*-RtcB_D94A_ mutant), which was subjected to extensive crystallization experiments in the presence of various RNAs. Despite the fact that crystals were obtained and approximately 100 datasets have been collected and analyzed, we did not observe any additional density corresponding to RNA in any of the solved crystal structures. Nonetheless, we decided to employ alternative approaches to gain insight into RNA binding of RtcB and in particular to map and identify the residues involved in RNA binding. We used a chemical cross-linking method based on the formation of a disulfide bond between a cysteine in the protein and an oligonucleotide in which the backbone phosphates of RNA are modified with cystamine (44).

As a starting point, we made predictions based on two types of structural information. First, in the crystal structures of nucleic acid–binding proteins, sulfate or phosphate anions often occupy pockets used by proteins to bind the phosphodiester backbone of RNA or DNA. Sulfate ions have been observed in several structures of *Pyrococcus horikoshii* RtcB (*Ph*-RtcB; Protein Data Bank [PDB] IDs: 4DWQ and 4ISJ). Second, a crystal structure of *Ph*-RtcB in complex with 6-mer DNA oligonucleotide mimicking the 5′ region of the 3′ exon is available (PDB ID: 7LFQ) (42). When sulfate-bound and DNA-bound structures were superimposed (PDB IDs: 4ISJ and 7LFQ, respectively), the phosphate groups of the second and third nucleotides of the DNA coincided with the position of sulfate ions in the 4ISJ structure (Fig. 1*B*). These analyses suggested the possible sites of the binding of phosphate groups of RNA. We decided to confirm whether the same sites that are involved in sulfate–DNA binding also participate in the interaction with the RNA backbone. For these studies, we used *Tb*-RtcB as the model enzyme. An AlphaFold 2 model (45) of *Tb*-RtcB was superimposed on the structure of *Ph*-RtcB–DNA complex (PDB ID: 7LFQ) (Fig. 1*C*). Analysis of the superposition indicated that three residues of *Tb*-RtcB could bind the nucleic acid backbone: L130, S199, and R237. The guanidinium group of R237 could form hydrogen bonds or ionic bonds with the phosphate group of the second and third nucleotides of the 3′ exon (numbered from 5′ end). This analysis also showed that S199 may form a hydrogen bond with the oxygen atom of the phosphate between the two first nucleotides, and L130 could form a van der Waals contact with the phosphate between the second and third nucleotides (Fig. 1*B*).

For chemical cross-linking reactions, we first prepared RtcB variants. We combined the active site mutant *Tb*-RtcB_D94A_ with three individual substitutions to cysteines resulting in variants: *Tb*-RtcB_D94A/L130C_, *Tb*-RtcB_D94A/S199C_, and *Tb*-RtcB_D94A/R237C_. Next, we designed two self-annealing RNA substrates that mimic the part of pre-tRNA molecule that is being ligated during catalytic reaction. We prepared two cystamine-modified version of this RNA: (i) RNA-1 with the cystamine attached to the phosphate of the phosphodiester bond between the second and third ribonucleotides from the 5′ end and (ii) RNA-2 with the cystamine localized on the phosphate between the first and second ribonucleotides. *Tb*-RtcB variants were mixed with these RNAs, and the reaction products were analyzed on nonreducing SDS-PAGE. We note that in our experiments a complex pattern of the cross-linking product bands is observed, but we focused our attention only on these products that are observed in the presence of RNA and thus do not result from protein–protein conjugation. We also note that protein–RNA crosslinks can exhibit anomalous migration on SDS-PAGE.

Specific protein–RNA crosslinks were observed between RNA-1 and all three substitution variants (L130C, S199C, or R237C) (Fig. 1*D*). For RNA-2, only the R237C mutant of *Tb*-RtcB formed specific cross-linking products (Fig. 1*E*). For the L130C and S199C *Tb*-RtcB variants, in the presence of RNA-2, only protein–protein conjugates were formed, as observed in the control experiment with no RNA oligonucleotide added (Fig. 1*E*). This was the case even when the DTT concentration was increased to 1 mM (Fig. 1*E*). Based on structural analyses, S199 is positioned closer to the phosphodiester bond between the first and second nucleotides, but its side chain is directed toward the phosphodiester bond between the second and third nucleotides. Crosslinking of S199C mutant with the RNA-2 substrate would result in a strained conformation (Fig. 1, *C* and *E*). This is why this variant forms crosslinks with RNA-1 substrate but not RNA-2. Obtained results showed that only certain combinations of cysteine variants and modified oligonucleotides formed cross-linked species, which confirms the specificity of the cross-linking reaction. Taken together, all these observations indicate that residues L130, S199, and R237 of *Tb*-RtcB interact with RNA. This suggests that the interactions between RNA and RtcB are very similar to those observed for the DNA mimic.

### tRNA-LC from *D. rerio* is stable and functional

**Figure 2:**
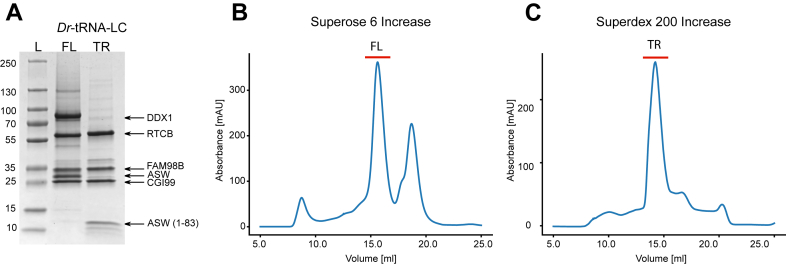
**Purification of the *Dr*-tRNA-LC.***A*, SDS-PAGE analysis of fractions from gel filtration. *B* and *C*, chromatograms from gel filtration. *Dr*-tRNA-LC, ligase complex from *Danio rerio*; FL, full-length version of *Dr*-tRNA-LC; L, molecular weight ladder; TR, truncated version of *Dr*-tRNA-LC.

To understand the architecture of the multisubunit tRNA-LC from metazoans, we decided to study the complex from *D. rerio* (*Dr*-tRNA-LC). We coexpressed all five components of *Dr*-tRNA-LC (DDX1, RTCB, FAM98B, CGI99, and ASW) using the BigBac insect cell expression system. We employed immobilized metal-affinity chromatography as the first step to purify the complex *via* His6–MBP fusion with DDX1, obtaining stable, homogeneous, and stoichiometric complex. Based on our initial structural studies, published crosslinking coupled to mass spectrometry analysis (41), and AlphaFold 3 predictions (see later), we also designed a truncated version (TR) of *Dr*-tRNA-LC, which comprised His6-RTCB, FAM98B, CGI99, the N-terminal part of ASW (residues 1–83), and the C terminus of DDX1 (residues 693–740). These complexes were further purified using gel filtration (Fig. 2).

**Figure 3:**
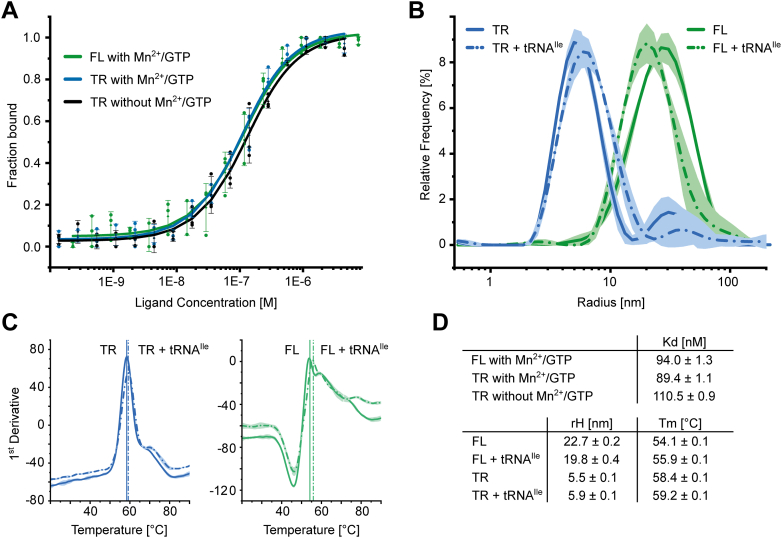
**Characterization of the purified FL and TR *Dr*-tRNA-LC.***A*, binding curves of titration of purified tRNA-LC with fluorescently labeled zebrafish tRNA^Ile^. Error bars indicate SD. n = 3 (independent dilution series, measurements of all three replicas are shown). *B*, size analysis by DLS of purified FL (*green*) and TR (*blue*) tRNA-LC in the presence or the absence of tRNA^Ile^. n = 5 (technical replicates). *C*, first derivative of thermal melting curves obtained for purified FL (*green*; *right*) and TR (*blue*; *left*) tRNA-LC in the presence or the absence of tRNA^Ile^. Inflection points used for *T*_*m*_ value calculation are indicated by *vertical lines*. n = 5 (technical replicates). *D*, quantification of the presented results. DLS, dynamic light scattering; *Dr*-tRNA-LC, ligase complex from *Danio rerio*; FL, full-length; rH, hydrodynamic radius; TR, truncated LC; tRNA-LC, tRNA ligase complex.

We next wanted to verify the functionality of both *Dr*-tRNA-LC preparations by testing their ability to bind *in vitro*–transcribed full-length (FL) *D. rerio* tRNA^Ile^, which represents the reaction product. We performed microscale thermophoresis (MST) analyses, which showed that tRNA^Ile^ interacts with FL (*K*_*d*_ = 94 ± 1.3 nM) as well as TR tRNA-LC (*K*_*d*_ = 89.4 ± 1.1 nM) with high affinity *in vitro* (Fig. 3, *A* and *D*). Of note, the binding of tRNA^Ile^ in the absence of Mn^2+^ and GTP is almost identical, and these cofactors seem to play only a minor role in the tRNA binding (Fig. 3, *A* and *D*). Next, we analyzed the purified samples by nano dynamic light scattering (nanoDLS) and nano differential scanning fluorimetry (nanoDSF). The results showed that both, the FL as well as the TR complex, were relatively thermostable and homogenous. However, the FL complex showed an unexpectedly large hydrodynamic radius of approximately 20 nm, indicating intrinsically highly dynamic parts of the assembly and/or the formation of its dimers or oligomers (Fig. 3, *B* and *D*). The addition of tRNA seemed to induce a slight compaction of the complex but did not dramatically change its overall size. The shortening of DDX1 and ASW in the TR complex led to the formation of a monomeric complex, and tRNA binding did not induce further compaction (Fig. 3, *B* and *D*). These observations are confirmed by an increased thermostability of the TR complex (*T*_*m*_ = 58.4 ± 0.1 °C) compared with the FL one (*T*_*m*_ = 54.1 ± 0.1 °C) (Fig. 3, *C* and *D*). The binding of tRNA did not increase the *T*_*m*_ values significantly, which indicated that the complex does not undergo any major structural rearrangements during tRNA binding. However, the results show that the helicase domain of DDX1 is dispensable for tRNA binding and that the C terminus of DDX1 is sufficient to form a stable core complex.

**Figure 4:**
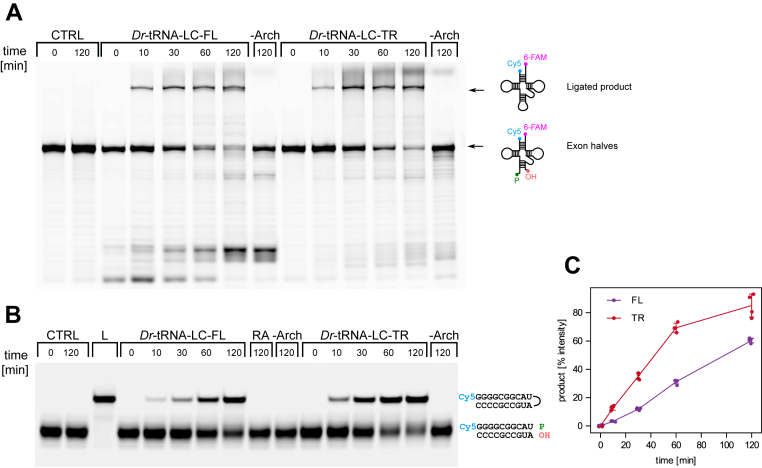
**Ligase activity of *Dr*-tRNA-LC.***A*, urea-PAGE analysis of the products of the enzymatic reaction carried out by the *Dr*-tRNA-LC (full-length [FL] and truncated [TR] variant) on the tRNA exon halves substrate. All reactions were performed in three replicates. *B*, urea-PAGE analysis of the products of the enzymatic reaction carried out by the *Dr*-tRNA-LC (FL and TR variant) on the short RNA substrate. Control reaction (CTRL) contained the RNA substrate and *Dr*-Archease but did not contain *Dr*-tRNA-LC. “–Arch” lane is the reaction without *Dr*-Archease. L, RNA corresponding to the expected product of the reaction. RA denotes the lane with R263A mutant. A schematic representation of the substrate and product is shown on the *right*. All reactions were performed in four replicates, (*C*) quantification of the data in (*B*). All four data points are plotted with error bar representing the SD of the measurements. *Dr*-tRNA-LC, ligase complex from *Danio rerio*.

We next performed enzymatic activity assays for *Dr*-tRNA-LC. Endonucleolytic cleavage of the pre-tRNA produces two tRNA exons. The 5′-exon with a 2′,3′-cyclic phosphate at the 3′-end and the 3′-exon with the hydroxyl group at the 5′-end. In the first step of the catalytic reaction, the hydrolysis of the cyclic phosphate results in the formation of a 3′-phosphate modification at the 3′-end of the 5′-exon. Thus, RNA containing a 3′-phosphate modification represents a good substrate for the ligase, as shown previously (17, 18). Therefore, we first used tRNA halves with 3′-phosphate and 5′-hydroxyl modifications to test ligase activity. In addition, the 5′-end of the 5′ exon was fluorescently labeled with Cy5 and 3′-end of the 3′ exon was labeled with fluorescein to monitor the product formation. RtcB requires Archease, a protein cofactor, for activity (23, 24, 25). Therefore, we added *D. rerio* Archease (*Dr*-Archease) to a subset of reactions. For the reaction with tRNA exon halves, we observed a product band for both FL and TR complexes (Fig. 4*A*). The product showed the signal from both Cy5 and fluorescein indicating that it was a proper ligation product (Cy5 scan is shown in Fig. 4*A*). *Dr*-tRNA-LC showed no activity when *Dr*-Archease was absent from the reaction.

The amount of the tRNA product could not be precisely quantified because of the RNA degradation. Therefore, we used a shorter 10-base pair (bp) RNA duplex, prepared by annealing two RNA strands: the first with a 3′-phosphate modification and fluorescently labeled at its 5′ end and the second with a 5′-hydroxyl group. For this substrate, we also observed a ligation product with the expected migration in the gel (Fig. 4*B*). Similar to the tRNA exon halves substrate, no reaction product was observed in the absence of Archease.

For the shorter substrate, the TR version of the complex catalyzed the ligation with higher efficiency than the FL counterpart, as evidenced by thicker bands corresponding to the reaction product (Fig. 4*B*). This was verified by quantification, which showed that ∼60% of the substrate was converted to the product for FL and ∼85% for TR after 120 min (Fig. 4*C*). These results suggest that the DDX1 subunit that is almost completely removed in the TR complex may have inhibitory effect on the activity of the complex. This is consistent with previous studies showing that the truncated human tRNA-LC exhibited higher ligase activity. In this case, a “minimal” four-subunit tRNA-LC, consisting of RTCB and the small C-terminal alpha-helical regions of DDX1, CGI99, and FAM98B, demonstrated enhanced activity compared with the FL counterpart (41).

### RTCB, FAM98B, CGI99, ASW, and C-terminal α-helix of DDX1 form a rigid part of the complex

To date, no structure is available for the entire tRNA-LC, and, therefore, the understanding of its molecular architecture remains incomplete. Such a structure would provide insight into catalytic mechanism of the complex and would help explain the role of additional subunits. We used single-particle cryo-EM to structurally characterize the purified *Dr*-tRNA-LC (Fig. S1). In 2D classes, we observed a rigid core region with clearly visible secondary elements, which was surrounded by a blurred region that probably corresponded to the flexible parts of the assembly (Fig. S1*D*). However, the overall resolution of the reconstruction was limited to 6.3 Å, most likely because of the preferential orientation of the particles on the grids (Fig. S1*E*). Several methods were used to alleviate the orientational bias, including the use of graphene oxide–layered grids and the addition of detergents. However, no significant improvement was observed. Due to the limited resolution, secondary structures could not be unambiguously identified in the cryo-EM map, but the overall shape of the *Dr*-tRNA-LC could be determined and analyzed (Fig. S1*F*).

We next used AlphaFold 3 to generate a model of the *Dr*-tRNA-LC (Figs. S1*F*, S3*A*) (46). This model was fitted into the obtained cryo-EM map. The part of the tRNA-LC complex, which comprised RTCB, FAM98B, CGI99, and the N-terminal part of ASW, could be fitted very well to our cryo-EM map (Fig. S1*F*). We did not observe density for the DDX1 subunit suggesting that it is a flexible element of the complex. This was further confirmed when 2D classes were simulated based on the AlphaFold 3 model of *Dr*-tRNA-LC. Those simulated 2D projections clearly contained additional density, which was not visible in the 2D classes from cryo-EM data, so it likely corresponded to DDX1 (Fig. S3, *A* and *B*). Similar 2D classes without the additional DDX1 signal were also observed for the samples of purified *Dr*-tRNA-LC mixed with tRNA substrate, although the quality of the data was not sufficient to obtain an interpretable 3D reconstitution (Fig. S3*C*).

These observations implied that DDX1 is a mobile part of the complex, so we designed a TR version of *Dr*-tRNA-LC, which comprised His6-RTCB, FAM98B, CGI99, the N-terminal part of ASW (residues 1–83), and the C terminus of DDX1 (693–740). For ASW, residues 1 to 83 were predicted to have an ordered helical N-terminal portion that interacts with the FAM98B–CGI99 heterodimer. The remaining portion of this subunit was predicted to form a long loop that did not interact with any other subunit of the complex, so we decided to remove it. For DDX1, we removed the mobile catalytic domain, but we kept the C-terminal region, which in the model it adopted an α-helical structure forming a bundle with α-helices of CGI99 and FAM98B.

**Figure 5:**
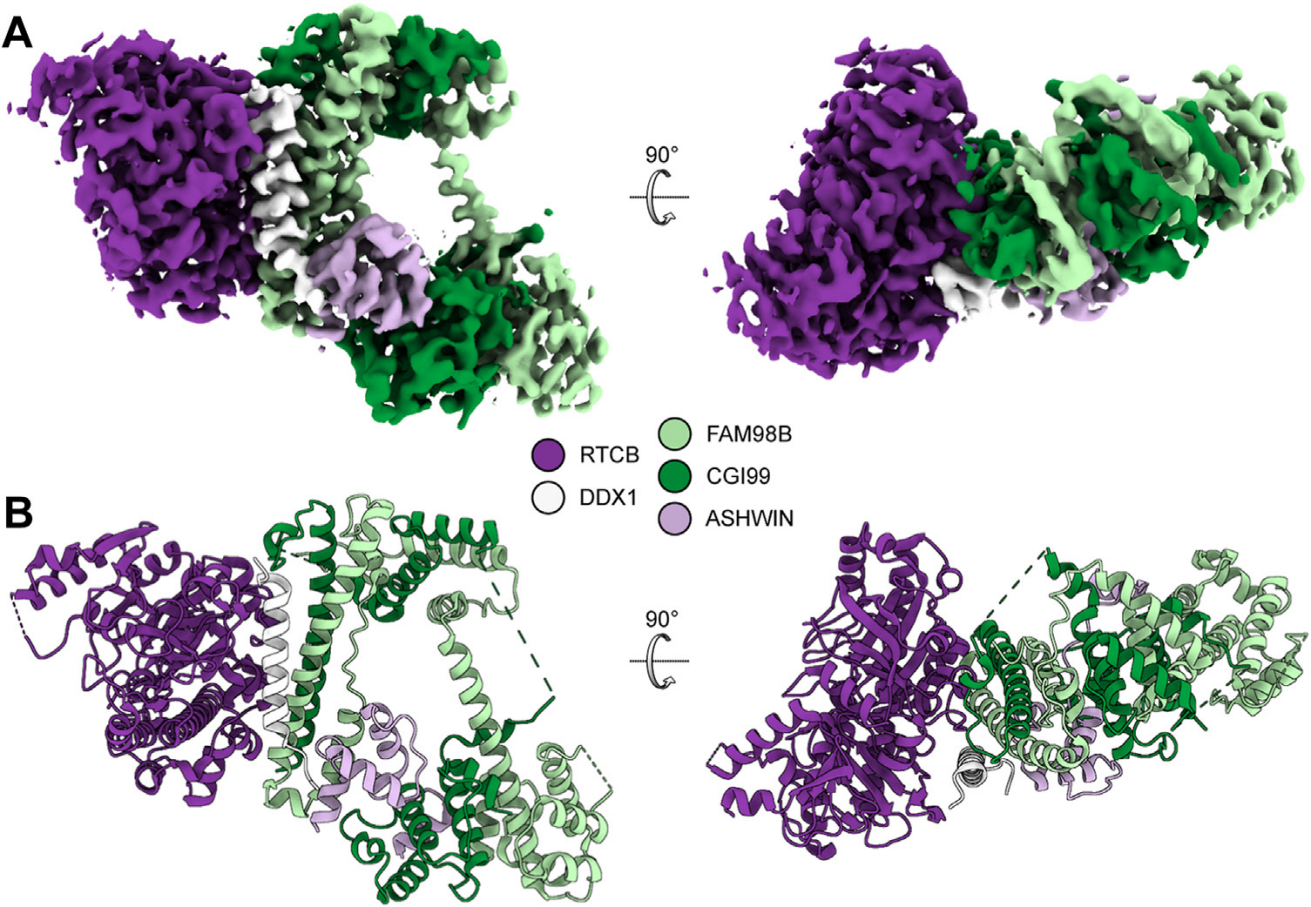
**Structure and model of *Dr*-tRNA-LC-TR.***A*, 3.3 Å cryo-EM reconstruction of *Dr*-tRNA-LC-TR. *B*, atomic model built based on the reconstruction. *Dr*-tRNA-LC, ligase complex from *Danio rerio*; TR, truncated version.

We next obtained a 3.3 Å reconstruction of *Dr*-tRNA-LC-TR (Figs. 5*A*, S2), which was used to build an atomic model of the complex (Fig. 5*B*). The structure was similar to the AlphaFold 3 prediction with rmsd of 1.8 Å over 964 C-α atoms. In the structure, the enzymatic subunit RTCB forms contacts with every other subunit of the complex except for ASW (Fig. 5). As predicted, the C-terminal helix of DDX1 is positioned between the RTCB subunit and the C-terminal α-helix of FAM98B. This is partly in agreement with a crosslinking coupled to mass spectrometry analysis, of the human tRNA-LC, where the C-terminal region of DDX1 formed many crosslinks with the other components of the five-subunit complex, with the exception of FAM98B (41). Moreover, this C-terminal helix has been shown to be the only region of DDX1 that is essential for the assembly of the complex (41).

In the structure, FAM98B and CGI99 form a highly intertwined heterodimer, as confirmed by our coexpression and purification of the human counterparts (His6-MBP-FAM98B and CGI99, Fig. S4). CGI99 eluted with FAM98B from nickel column, even though it did not contain the His6 tag, which indicated a stable interaction of these two subunits also outside the fully assembled tRNA-LC complex. Similar observations were made using coprecipitation assays (41). In addition, FAM98B was soluble only in the presence of CGI99, suggesting a stabilizing effect of CGI99 on FAM98B. In our structure, the C-terminal helical portions of CGI99 and FAM98B dimer interact with the α-helical region of RTCB (residues 340–393). The same part of the CGI99–FAM98B heterodimer binds the ordered N-terminal region of ASW. This observation is supported by previous results from affinity purification experiments, which showed that in the absence of either DDX1 or RTCB, ASW formed a complex with the CGI99–FAM98B dimer but not with either of these subunits alone (41). In addition, cross-linking coupled to mass spectrometry analysis revealed numerous crosslinks between ASW and all other complex subunits (41). Perhaps the unstructured C-terminal region of ASW transiently interacts with the other proteins in the complex.

In some of the cryo-EM samples, including the one that was used for the high-resolution *Dr*-tRNA-LC-TR structural studies, we included *in vitro*–transcribed tRNA^Leu^ or tRNA^Ile^. However, in none of the reconstructions, we could observe a well-defined density for the RNA. So, we decided to use biochemical assays to verify whether *Dr*-tRNA-LC exhibits the same mode of RNA binding as identified by our experiments with *Tb*-RtcB. Our chemical cross-linking results showed that for the archaeal protein the residue important for RNA binding is R237, which can interact with two consecutive phosphate groups of the RNA. Its equivalent in *Dr*-RTCB is R263. We prepared a variant of *Dr*-tRNA-LC-FL with R263A substitution. This variant was completely devoid of enzymatic activity confirming the importance of R263 for RNA binding (Fig. 4*B*).

Taken together, our structural data show the arrangement of the subunits of the tRNA-LC. CGI99–FAM98B form an intertwined dimer, and N-terminal domain of ASW, C-terminal helix of DDX1, and catalytic subunit RTCB interact with a centrally located helical bundle formed by CGI99 and FAM98B.

### DDX1 is a mobile subunit of the complex, which affects the ligase activity *in vitro*

In the AlphaFold 3 model of the FL *Dr*-tRNA-LC, the N-terminal part of DDX1 (RecA-like domain 1) forms multiple contacts with RTCB, especially its C-terminal region. The SPRY domain of DDX1 is distant from the entire assembly, as is the C-terminal part of the protein, with the exception of the C-terminal α-helix (Fig. S3*A*). We compared the medium-resolution reconstructions of FL *Dr*-tRNA-LC with the higher resolution map for the TR variant (Fig. S3, *B*–*D*). These comparisons showed that the overall shape of these reconstructions is very similar, which implied that the density for the DDX1 helicase domain is not visible in the reconstruction of the FL complex. Moreover, a blurred region in 2D classes of the *Dr*-tRNA-LC-FL was observed, which likely corresponds to mobile parts of DDX1 (Fig. S3*B*). This observation is supported by the nanoDLS and nanoDSF analyses, showing that the FL complex is less stable/more dynamic than the TR complex (Fig. 3*B*). In addition, the unexpectedly large hydrodynamic radius of the FL complex could be explained by the mobility of the helicase domain of DDX1.

**Figure 6:**
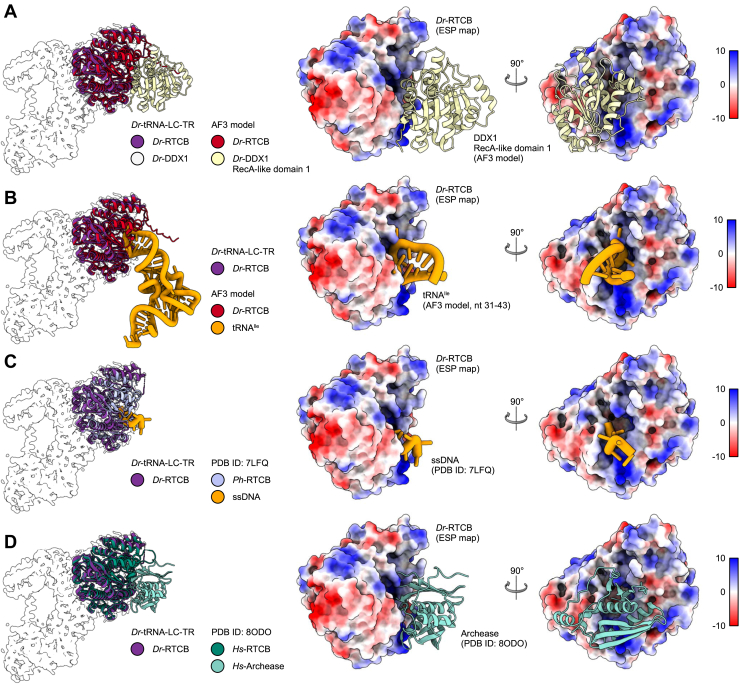
**The position of DDX1 subunit in *Dr*-tRNA-LC.***A*, superimposition of the AlphaFold 3 model of the *Dr*-tRNA-LC-FL on *Dr*-RTCB from the *Dr*-tRNA-LC-TR structure. *Left*, *Dr*-RTCB and *Dr*-DDX1 shown on the contour of the *Dr*-tRNA-LC-TR map. *Right*, modeled interaction between DDX1 RecA-like domain 1 (AlphaFold 3 model) and *Dr*-RTCB (shown as Coulombic electrostatic potential surface); two views. *B*, superimposition of the AlphaFold 3 model of the *Dr*-tRNA-LC-TR-tRNA^Ile^ on *Dr*-RTCB from the *Dr*-tRNA-LC-TR structure. *Left*, *Dr*-RTCB and tRNA^Ile^ shown on the contour of the *Dr*-tRNA-LC-TR map. *Right*, modeled interaction between tRNA^Ile^ (AlphaFold 3 model; only anticodon loop is shown for clarity) and *Dr*-RTCB (shown as Coulombic electrostatic potential surface); two views. *C*, superimposition of *Ph*-RtcB in a complex with ssDNA (PDB ID: 7LFQ) on *Dr*-RTCB from the *Dr*-tRNA-LC-TR structure. *Left*, *Dr*-RTCB, *Ph*-RTCB, and ssDNA shown on the contour of the *Dr*-tRNA-LC-TR map. *Right*, modeled interaction between ssDNA and *Dr*-RTCB (shown as Coulombic electrostatic potential surface); two views. *D*, superimposition of *Hs*-RTCB in complex with Archease (PDB ID: 8ODO) on *Dr*-RTCB from the *Dr*-tRNA-LC-TR structure. *Left*, *Dr*-RTCB, *Hs*-RTCB, and *Hs*-Archease shown on the contour of the *Dr*-tRNA-LC-TR map. *Right*, modeled interaction between Archease and *Dr*-RTCB (shown as Coulombic electrostatic potential surface); two views. *Dr*-tRNA-LC, ligase complex from *Danio rerio*; FL, full length; *Hs*, Homo sapiens; PDB, Protein Data Bank; *Ph*-RtcB, *Pyrococcus horikoshii*; TR, truncated version.

Interestingly, in the AlphaFold 3 model of the FL *Dr*-tRNA-LC, the N-terminal catalytic domain of DDX1 interacts with a region of RTCB, which is located near its active site (Fig. 6*A*). Using thiol-based chemical crosslinking, we confirmed that the corresponding region in archaeal RtcB binds RNA. We also showed that the DNA chain from the structure of the *Ph*-RtcB–DNA complex (PDB ID: 7LFQ) (42) is a good mimic of the RNA binding (Fig. 1, *B* and *C*). Moreover, analysis of the electrostatic potential of the surface of the *Dr*-tRNA-LC-TR (based on our cryo-EM structure) revealed positively charged patches, located near the modeled position of the tRNA^Ile^ molecule, as predicted by AlphaFold 3 in the absence of DDX1 subunit (Fig. 6). These patches could be responsible for RNA binding and may be the main RNA-binding interface. This means that most of the tRNA would not interact with the protein, which would result in its high mobility, explaining why it could not be visualized in our cryo-EM potential maps.

We also superimposed the *Ph*-RtcB–DNA complex on the RTCB subunit of the model of *Dr*-tRNA-LC. In this superposition, the DNA chain from *Ph*-RtcB complex and the DDX1 subunit in the AlphaFold 3 model coincided (Fig. 6, *A* and *C*). Furthermore, we also compared the LC model with the structure of RTCB–Archease complex (PDB ID: 8ODO) (26). Here, Archease and DDX1 also formed steric clashes (Fig. 6, *A* and *D*). This suggests that DDX1 could exhibit regulatory effect by restricting the access of Archease and/or the RNA substrate to the active site of RTCB. This is in agreement with lower ligase activity of the FL complex comprising DDX1 compared with the TR form, which lacked it (Fig. 4, *B* and *C*). We also showed that the affinities of the FL and TR complexed for the tRNA product were essentially indistinguishable (Fig. 3*A*). This shows that DDX1 does not modulate RNA binding. The fact that its presence reduces enzymatic activity is thus probably because of its interference with the binding of the Archease. Collectively, these results show that the mobile DDX1 subunit has a negative effect on the activity of the complex. The elucidation of the exact functional role of this mechanism will require further studies.

It has been speculated that the noncatalytic subunits of tRNA-LC may negatively regulate the catalytic activity of RTCB (41). We showed that the presence of DDX1 inhibited the activity of tRNA-LC. It has also been shown that the activity of the complex was not increased in the presence of ATP (41), which is in agreement with the results presented herein. Interestingly, another study showed that DDX1 in combination with Archease increased the ligase activity of RTCB (27). These results imply that the DDX1 may have a complex influence on the tRNA LC activity, perhaps also depending on the type of RNA substrate it acts upon, and the full elucidation of its role awaits further studies.

## Discussion

tRNA ligases play a central role in the maturation of specific tRNAs, which are essential elements of protein synthesis. Despite the importance of tRNA ligases, their exact mode of tRNA substrate binding and the architecture of the higher eukaryotic tRNA-LC were not fully characterized. Here, we show that three RtcB residues interact with the first two phosphates of the 3′ exon, verifying the RNA binding mode inferred from previous structural data visualizing the binding of sulfate ions and a DNA mimic by archaeal RtcB. This information helps to identify the RNA substrate trajectory and can be used to model RtcB–substrate interactions and to design future biochemical, structural, and functional studies of the ligase–tRNA complexes.

Our second goal was to provide structural information for the five-subunit tRNA-LC in animals. We purified the *Dr*-tRNA-LC and reconstituted its tRNA ligase activity in the presence of the Archease cofactor *in vitro*. We obtained a medium-resolution single-particle cryo-EM reconstruction of the FL complex and a 3.3 Å structure of its TR version lacking the catalytic domain of DDX1 and unstructured region of ASW. We thus provide the first structural information on the architecture of the fully assembled tRNA-LC. One key insight from this work is that the DDX1 component of the complex is mobile. It can regulate the access to the active site, and our biochemical experiments imply that it may be reducing the Archease binding in the ligase activation step. These results provide a framework for further detailed structural and mechanistic studies of the tRNA-LC. It will now be important to confirm the mechanism of regulation of the tRNA-LC activity by DDX1 component and identify its physiological role.

## Experimental procedures

### Expression and purification of *Tb*-RtcB

The synthetic gene that encodes wt *Tb*-RtcB was subcloned into a pET-28 expression vector that carried an N-terminal His6-SUMO tag, which can be removed by SUMO protease. *Tb*-RtcB wt and respective mutants were overproduced in *Escherichia coli* BL21 Star (DE3) cells in Lysogen broth medium after induction with 0.6 mM isopropyl β-D-1-thiogalactopyranoside at 18°C. The cells were then harvested and suspended in the lysis buffer (25 mM Hepes [pH 7.5], 200 mM NaCl, 5% glycerol, 10 mM imidazole, 10 mM β-mercaptoethanol, and 5 mM MnCl_2_) and incubated on ice in the presence of lysozyme (1 mg/ml), protease inhibitor cocktail, and viscolase. After sonication and centrifugation at 185,700*g* for 40 min at 4 °C, the cleared lysate was applied to a HisTrap column (Cytiva) that was equilibrated with buffer A (25 mM Hepes [pH 7.5], 500 mM NaCl, 5% glycerol, 10 mM imidazole, and 5 mM β-mercaptoethanol). After a wash step with buffer A supplemented with 60 mM imidazole, the protein was eluted with the same buffer containing 300 mM imidazole. The eluted fraction was dialyzed overnight against dialysis buffer (25 mM Hepes [pH 7.5], 500 mM NaCl, 5% glycerol, and 5 mM β-mercaptoethanol) in the presence of SUMO protease to remove the His6-SUMO tag and subsequently reapplied onto a HisTrap column that was equilibrated with buffer A. The flow-through fraction that contained cleaved protein was pooled, concentrated, and further purified on a Superdex 200 Increase 10/300 GL column (Cytiva) that was equilibrated with the GF buffer (25 mM Hepes [pH 7.5], 250 mM NaCl, 5% glycerol, and 1 mM DTT). Selected fractions were concentrated, flash frozen in liquid nitrogen, and stored for further use.

### Chemical crosslinking

Expression constructs for *Tb*-RtcB variants for cross-linking experiments (*Tb*-RtcB_D94A/L130C_, *Tb*-RtcB_D94A/S199C_, and *Tb*-RtcB_D94A/R237C_) were prepared according to Stratagene’s QuikChange protocol, and the proteins were purified as wt, with the exception that the GF buffer lacked DTT.

Cross-linking reactions contained 3.5 μM *Tb*-RtcB, 8.75 μM substrate (either RNA-1 [5′ A(A)∗ACACCGGUGGAGG-P 3′] or RNA-2 [5′ (A)∗AACACCGGUGGAGG-P 3′], where ∗ denotes the cystamine modification, (A) denotes nucleotide with fluorine atom at the 2′ ribose position, and P denotes phosphate; synthesized by FutureSynthesis), 50 mM Tris (pH 7.5), 25 mM NaCl, 25 mM KCl, 30% glycerol, and 2.5 mM MnCl_2_ and were incubated at 37 °C for 2 h, followed by 14 h at 24 °C. Both oligonucleotides contained a 2′-F modification next to the phosphate modification. We have previously shown that this modification is required to ensure the stability of cystamine-modified RNA (44). A concentration range of 0 to 1 mM DTT was tested to optimize the experimental protocol. Cross-linked samples were analyzed on 4 to 15% gradient Mini-PROTEAN TGX Precast Protein Gels (Bio-Rad) and visualized with Coomassie blue stain.

### Expression and purification of *Homo sapiens* FAM98B–CGI99 heterodimer

The genes encoding the *H. sapiens* CGI99 (*Hs*-CGI99) and *H. sapiens* FAM98B (*Hs*-FAM98B) were cloned into a pFastBac Dual vector with an N-terminal His6-MBP tag in fusion with *Hs*-FAM98B, which can be removed by tobacco etch virus (TEV) protease. Recombinant baculoviruses were prepared in the Bac-to-Bac system (Invitrogen) using standard procedures. Constructs were expressed in Sf9 insect cells grown in suspension. For protein expression, cells were infected with the second generation of virus at a cell density of 2 to 2.5 × 10^6^ cells/ml. Cells were grown for 72 h postinfection and then collected and centrifuged. Pellets were flash frozen in liquid nitrogen and stored at −20 °C.

For protein purification, the pellets were suspended in the lysis buffer (25 mM Hepes [pH 7.5], 100 mM NaCl, 5% glycerol, 20 mM imidazole, 5 mM MgCl_2_, and 5 mM β-mercaptoethanol) supplemented with protease inhibitor cocktail and viscolase and incubated on ice for 30 min. Following sonication and centrifugation at 185,700*g* for 40 min at 4 °C, the cleared lysate was applied to a HisTrap column that was equilibrated with buffer A (25 mM Hepes [pH 7.5], 100 mM NaCl, 5% glycerol, 20 mM imidazole, and 5 mM β-mercaptoethanol). After a wash step with buffer A supplemented with 60 mM imidazole, the protein was eluted with the same buffer containing 300 mM imidazole. Eluted fraction was dialyzed overnight against the dialysis buffer (25 mM Hepes [pH 7.5], 100 mM NaCl, 5% glycerol, and 5 mM β-mercaptoethanol) in presence of TEV protease to remove the His6-MBP tag and reapplied onto a HisTrap column equilibrated with buffer A. Selected elution fractions were pooled, concentrated, analyzed by SDS-PAGE, and visualized with Coomassie blue stain.

### Expression and purification of *Dr*-tRNA-LC (FL and TR forms)

Genes encoding all five subunits of *Dr*-tRNA-LC: DDX1 (fusion with His6-MBP), RTCB, FAM98B, CGI99, and ASW were cloned into a pBIG1a vector. The same vector was used to prepare a construct for the expression of a TR version of the complex comprising FL His-tagged RTCB, FAM98B, CGI99, and deletion variants of DDX1 (residues 693–740) and ASW (residues 1–83). Recombinant baculoviruses were prepared in the Bac-to-Bac system (Invitrogen) using standard procedures. Genes were expressed in Sf9 insect cells grown in suspension. For gene expression, cells were infected with the third generation of virus at a cell density of 2 to 2.5 × 10^6^ cells/ml. Cells were grown for 72 h postinfection and then collected and centrifuged. Pellets were flash frozen in liquid nitrogen and stored at −20 °C.

For protein purification, the pellets were suspended in the lysis buffer (25 mM Hepes [pH 7.5], 150 mM NaCl, 5% glycerol, 20 mM imidazole, 5 mM MgCl_2_, and 5 mM β-mercaptoethanol) and incubated on ice in the presence of protease inhibitor cocktail and viscolase. Following sonication and centrifugation at 6493*g* for 60 min at 4 °C, the cleared lysate was applied to a HisTrap column equilibrated with buffer A (25 mM Hepes [pH 7.5], 150 mM NaCl, 5% glycerol, 20 mM imidazole, and 5 mM β-mercaptoethanol). After a wash step with buffer A supplemented with 60 mM imidazole, the proteins were eluted with the same buffer containing 300 mM imidazole. Selected fractions of FL protein were dialyzed overnight against 20 mM imidazole, 25 mM Hepes [pH 7.5], 150 mM NaCl, 5% glycerol, and 5 mM β-mercaptoethanol in the presence of TEV protease to remove His6-MBP tag. Samples were then reapplied to a HisTrap column under the same conditions. Cleaved protein did not bind to the column and was collected in the flow-through. FL complex was further purified on a Superose 6 Increase 10/300 GL column (Cytiva).

In order to remove nucleic acid contamination, the TR complex purified from the first HisTrap column step was applied to a heparin column (Cytiva) and equilibrated with heparin buffer (25 mM Hepes [pH 7.5], 150 mM NaCl, 5% glycerol, and 1 mM DTT). Protein was eluted using a linear gradient of 150 to 1000 mM NaCl. Fractions containing the highest purity protein were pooled and injected onto Superdex 200 Increase 10/300 GL column and equilibrated with GF buffer (25 mM Hepes [pH 7.5], 150 mM NaCl, and 1 mM DTT). Selected fractions were concentrated, flash frozen in liquid nitrogen, and stored at −20 °C.

### Expression and purification of Archease

The synthetic ORFs that encode *Dr*-Archease was subcloned into a pET-28 expression vector that carried an N-terminal His6-SUMO tag that is removable by SUMO protease. Proteins were produced in *E. coli* BL21 Star (DE3) cells in Lysogen broth medium after induction with 0.4 mM isopropyl β-D-1-thiogalactopyranoside at 18 °C. The cells were then harvested and suspended in the lysis buffer (40 mM Tris–HCl [pH 7.0], 250 mM NaCl, 5% glycerol, 10 mM imidazole, and 10 mM β-mercaptoethanol) and incubated on ice in the presence of 1 mg/ml lysozyme, protease inhibitor cocktail, and viscolase. Following sonication and centrifugation at 185,700*g* for 40 min at 4 °C, the cleared lysate was applied to a HisTrap column equilibrated with buffer A (40 mM Tris–HCl [pH 7.0], 250 mM NaCl, 5% glycerol, 10 mM imidazole, and 5 mM β-mercaptoethanol). After a wash step with buffer A supplemented with 60 mM imidazole, the protein was eluted with the same buffer containing 300 mM imidazole. The eluted fraction was dialyzed overnight against the dialysis buffer (40 mM Tris–HCl [pH 7.0], 250 mM NaCl, 5% glycerol, and 5 mM β-mercaptoethanol) in the presence of SUMO protease to remove the His6-SUMO tag and reapplied onto a HisTrap column equilibrated with buffer A. The flow-through fraction that contained cleaved protein was collected, concentrated, and further purified on a Superdex 200 Increase 10/300 GL column equilibrated with the GF buffer (40 mM Tris–HCl [pH 7.0], 250 mM NaCl, 5% glycerol, and 1 mM DTT). Selected fractions were concentrated, flash frozen in liquid nitrogen, and stored at −20 °C.

### tRNA ligation activity assay

*In vitro* RNA ligase assays were performed at 37 °C at various time points. Fluorescently labeled RNA substrate was designed based on previous work (47). The substrate was prepared by annealing two RNA oligonucleotides: pre-tRNA1 (5′ Cy5-GCGGAUUUAGCUCAGUUGGGAGAGCGCCAGACUCCAG-P 3′) and pre-tRNA2 (5′ AUCUGGAGGUCCUGUGUUCGAUCCACAGAAUUCGCACCA-6-FAM 3′) (FutureSynthesis) at 1:1 M ratio. The substrate was added to the buffer (20 mM Tris–HCl [pH 8.0], 50 mM KCl, 5% glycerol, 2 mM Tris(2-carboxyethyl)phosphine hydrochloride, 100 μM GTP, and 1 mM MnCl_2_) to a final concentration of 0.15 μM. Subsequently, *Dr*-tRNA-LC (FL or TR form) was added at a final concentration of 0.6 μM. In a subset of reaction mixtures, *Dr*-Archease was added at a final concentration of 0.6 μM.

The short RNA substrate was prepared by annealing two RNA oligonucleotides: RNA_B (5′ AUGCCGCCCC 3′) and RNA_C (5′ Cy5-GGGGCGGCAU-P 3′) (Eurofins) at 1.1:1 molar ratio. The substrate was added to the buffer (20 mM Tris–HCl [pH 8.0], 150 mM NaCl, 5% glycerol, 2 mM Tris(2-carboxyethyl)phosphine hydrochloride, 0.001% Triton 100, 500 μM GTP, and 1 mM MnCl_2_) to a final concentration of 0.15 μM. *Dr*-tRNA-LC (FL or TR form) was then added to a final concentration of 0.15 μM. In a subset of reaction mixtures, *Dr*-Archease was added at a final concentration of 0.6 μM. The products were analyzed on 20% TBE–urea polyacrylamide gel and visualized by fluorescence readout using the Amersham Typhoon RGB Biomolecular Imager (Cytiva). Signal was quantified with ImageQuant TL software and normalized to the total RNA loaded to each lane.

### Glutaraldehyde crosslinking in glycerol gradient and cryo-EM sample preparation

To ensure proper stoichiometry and to stabilize the *Dr*-tRNA-LC-FL, the GraFix method was used. Briefly, GraFix is a density gradient centrifugation process that combines a glycerol gradient for size fractionation and fixation of macromolecular complexes by a mild chemical cross-linking agent (48, 49). The protein sample (1 mg/ml, 150 μl) was applied at the top of the tube with a 5 to 30% (v/v) glycerol gradient (25 mM Hepes [pH 7.5], 150 mM NaCl, 1 mM DTT, 0.1% glutaraldehyde, and glycerol) and centrifuged for 18 h at 183,959*g* at 4 °C. Fractions of 200 μl were then collected starting from the top of the gradient, and the cross-linking reaction was stopped by the addition of Tris–HCl (pH 8.0) to a final concentration of 50 mM. The quality of the fractions used for cryo-EM sample preparation was assessed by SDS-PAGE. Before cryo-EM grid preparation, glycerol was removed by buffer exchange (25 mM Hepes [pH 7.5], 150 mM NaCl, and 1 mM DTT) using Amicon Ultra-4 (100 kDa cutoff; Millipore). Cryo-EM sample of *Dr*-tRNA-LC-TR was prepared in the following way: the protein sample at concentration of 0.95 mg/ml was mixed with MnCl_2_ and GTP (both at final concentrations of 2 mM). Subsequently, twofold molar excess of tRNA^Ile^ (5′ GGCUCCAGUGGCGCAAUCGGUUAGCGCGCGGUACUUAUAAUGCCGAGGUUGUGAGUUCGAGCCUCACCUGGAGCACCA 3′) was added, and the sample was incubated for 15 min at room temperature. Samples (3 μl) were vitrified in liquid ethane on glow-discharged Quantifoil 2/1 mesh 200 Cu (*Dr*-tRNA-LC-FL, 0.4 mg/ml) or C-flat 2/1 mesh 200 Cu (*Dr*-tRNA-LC-TR, ∼0.90 mg/ml) grid using an FEI Vitrobot Mark IV (Thermo Fisher Scientific) at 4 °C and 95% humidity, and with a 4 s blot time, 0 s wait time, and 0 blot force. Data collection was performed on a Titan Krios G3i cryo-electron microscope (Thermo Fisher Scientific) operating at 300 kV and equipped with a BioQuantum energy filter (set to 20 eV energy slit) and K3 direct electron detector (Gatan), at the SOLARIS National Synchrotron Radiation Centre in Krakow, Poland. Raw movies were collected with aberration-free image shift, at a nominal magnification of 105,000×. See Table S1 for data collection parameters.

### Cryo-EM data processing

#### *Dr*-tRNA-LC-FL

Cryo-EM data were processed with RELION-3.0 (Medical Research Council Laboratory of Molecular Biology) (50) and cryoSPARC 3.3 (Structura Biotechnology Inc) (51) (Fig. S1). A total of 10,703 movies were motion-corrected and binned 2× using RELION’s implementation of MotionCor2 software (University of California, San Francisco [UCSF]) (52). After motion correction, the contrast transfer function (CTF) estimation was performed using CTFFIND 4.1 (53) to fit the CTF. Next, crYOLO (Max-Planck Institute of Molecular Physiology, Group Raunser, Thorsten Wagner) (54), an application for fast and accurate cryo-EM particle picking was used to pick 1,316,517 particles, which were further extracted with the box size of 160 pixels (54). Particles were imported to cryoSPARC and subjected to several rounds of 2D classification. A total of 872,772 selected particles were used to generate three initial 3D models. Selected classes were subjected to additional rounds of 2D classification to finally select 37,015 particles. Next, the particles were subjected to homogeneous refinement. Refined particles were next reimported into RELION using PyEM (55) script and subjected to Bayesian polishing (with re-extraction with an unbinned pixel size of 0.86 Å/pixel), followed by another round of 2D classification in cryoSPARC and final selection of 35,251 particles. To improve the quality of the reconstruction, a mask covering the rigid part of the reconstruction was generated in UCSF Chimera (56) software and used for local refinement in cryoSPARC.

#### *Dr*-tRNA-LC-TR

Cryo-EM data were processed with RELION-5.0 (57) and cryoSPARC 4.6 (51) (Fig. S2). A total of 9942 movies (collected with a stage tilt of 20°) were motion-corrected and binned 2× using RELION’s implementation of MotionCor2 software (52). Initial CTF estimation was performed using CTFFIND 4.1 (53). A total of 4,812,967 particles were selected with crYOLO software, version 1.9.6 (54), extracted with 2× binning in a 80 pixel box in RELION, and processed in cryoSPARC to generate 2D templates and initial 3D references. The motion-corrected micrographs were imported into cryoSPARC, and CTF estimation was performed by Patch CTF. A total of 13,964,718 particles were picked by template picker and extracted with 2× binning in a box size of 80 pixel. To avoid eliminating rare poses, the 2D classification step was omitted and suboptimal and junk particles were eliminated with 22 iterative rounds of reference-based 3D classification by heterogeneous refinement with one good and four bad (decoy) 3D references. In between these classifications, the selected particles were subjected to unbinning and two rounds of Bayesian polishing in RELION as well as CTF refinements in cryoSPARC (Fig. S2*C*). The final set of particles was subjected to a second round of CTF refinements and to a local refinement in cryoSPARC, which resulted in a 3.33 Å reconstruction. This cryo-EM map was locally sharpened using the Local Filter tool in cryoSPARC and used for the model building of *Dr*-tRNA-LC-TR in Coot (MRC Laboratory of Molecular Biology [LMB]) (58), with the help of ModelAngelo software (Kiarash Jamali) (59) and the AlphaFold 3 model (46). This model was further subjected to multiple rounds of model building in Coot and real space refinement in Phenix (PHENIX Consortium) (60) (Table S1). The directional Fourier shell correlation was calculated by the 3D-FSC web server (61).

### MST of *Dr*-tRNA-LC and tRNA

The binding affinity between FL and TR *Dr*-tRNA-LC and tRNA^Ile^ (5′ GGCUCCAGUGGCGCAAUCGGUUAGCGCGCGGUACUUAUAAUGCCGAGGUUGUGAGUUCGAGCCUCACCUGGAGCACCA 3′) was measured by MST. The reaction was performed in buffer A (25 mM Hepes [pH 7.5], 150 mM NaCl, and 1 mM DTT) and in buffer B (buffer A supplemented with 2 mM GTP and 2 mM MnCl_2_). A series of 16 1:1 dilutions of *Dr*-tRNA-LC were prepared starting at 7.6 μM. The *Dr*-tRNA-LC dilutions were then mixed in a 1:1 ratio with 40 nM solutions of the Cy5-labeled tRNA^Ile^. The samples were loaded into capillaries (MO-K025), and the measurements were performed using Monolith 2020 (TNG, MM-018; NanoTemper Technologies), based on the fluorescent signal from the Cy5-labeled tRNA^Ile^. The data were analyzed using NanoTemper Analysis software (NanoTemper), and the *K*_*d*_ was calculated.

### NanoDLS

Samples for DLS analysis were prepared by diluting FL and TR *Dr*-tRNA-LC in buffer A (25 mM Hepes [pH 7.5], 150 mM NaCl, and 1 mM DTT) or buffer B (buffer A supplemented with 1 mM GTP and 1 mM MnCl_2_) to a concentration of 0.3 mg/ml. The protein samples were loaded into the high-sensitivity capillaries (PR-C006), and the measurement was performed using the Prometheus Panta (NanoTemper Technologies). Samples were prepared in triplicate. Datasets were analyzed using Panta Stability Analysis software (NanoTemper Technologies).

### NanoDSF

Thermal unfolding experiments were performed using Prometheus Panta. Protein samples were prepared in a buffer containing 25 mM Hepes (pH 7.5), 150 mM NaCl, 1 mM DTT, 2 mM GTP, and 2 mM MnCl_2_.

The final protein concentration was 0.5 mg/ml for both, the TR complex (133 kDa, extinction coefficient = 0.801) and the FL complex (223.5 kDa, extinction coefficient = 0.843). tRNA^Ile^ was added at a 5:1 M excess relative to the protein. Ten microliters of each sample were loaded into high-sensitivity capillaries (NanoTemper Technologies; PR-C006). Thermal unfolding was induced by applying a temperature gradient from 20 °C to 95 °C at a rate of 1 °C/min. Intrinsic protein fluorescence was monitored at 330 nm. The thermal stability of the samples was analyzed by calculating the first derivative of fluorescence intensity at 330 nm, and the respective *T*_*m*_ were determined as the temperature corresponding to the maximum slope of the fluorescence change. Measurements were performed for both the FL and TR protein forms, with and without tRNA, five replicates per condition were used to ensure reproducibility, and SDs were calculated for each of the obtained *T*_*m*_ values.

## Data availability

The cryo-EM map of *Dr*-tRNA-LC-FL has been deposited in the Electron Microscopy Data Bank under the accession code EMD-50108. The atomic model and corresponding cryo-EM map of *Dr*-tRNA-LC-TR has been deposited in the PDB and Electron Microscopy Data Bank under the accession codes PDB ID: 9I8V, EMD-52744. All other relevant data are available from the corresponding author.

## Supporting information

This article contains supporting information (61).

## Conflict of interest

The authors declare that they have no conflicts of interest with the contents of this article.
