# Supplementary material for: Structural and biochemical characterization of the 3′-5′ tRNA splicing ligases
Source: J Biol Chem. 2025 Apr 10;301(5):108506. doi: 10.1016/j.jbc.2025.108506 (PMC12135372; doi:10.1016/j.jbc.2025.108506)
Supplement: Supporting information [file mmc1.pdf]

Structural and biochemical characterization of the 3'-5' tRNA splicing ligases

Sebastian Chamera[1], Weronika Zajko[1], Mariusz Czarnocki-Cieciura[1], Marcin Jaciuk[2], Łukasz Koziej[2], Jakub Nowak[2], Krzysztof Wycisk[1], Małgorzata Sroka[1], Andrzej Chramiec-Głąbik[2], Mirosław Śmietański[1,3], Filip Gołębiowski[1], Marcin Warmiński[4], Jacek Jemielity[5], Sebastian Glatt[2,6], and Marcin Nowotny[1,*]

1. Laboratory of Protein Structure, International Institute of Molecular and Cell Biology, Warsaw, Poland

2. Malopolska Centre of Biotechnology (MCB), Jagiellonian University, Krakow, Poland

3. Explorna Therapeutics sp. z o.o., Warsaw, Poland

4. Division of Biophysics, Institute of Experimental Physics, Faculty of Physics, University of Warsaw, Warsaw, Poland

5. Centre of New Technologies, University of Warsaw, Warsaw, Poland

6. Department for Biological Sciences and Pathobiology, University of Veterinary Medicine Vienna, 1210 Vienna, Austria

SUPPORTING INFORMATION

Supplementary Figure 1. ***Dr*-tRNA-LC-FL cryo-EM data processing pipeline.**

Supplementary Figure 2. ***Dr*-tRNA-LC-TR cryo-EM data processing pipeline.**

Supplementary Figure 3. **Comparison of *Dr*-tRNA-LC models, maps, and 2D classes.**

Supplementary Figure 4. **Purification of the human FAM98B–CGI99 heterodimeric complex from insect cells.**

Supplementary Table 1. **Cryo-EM data collection, refinement, and validation statistics.**

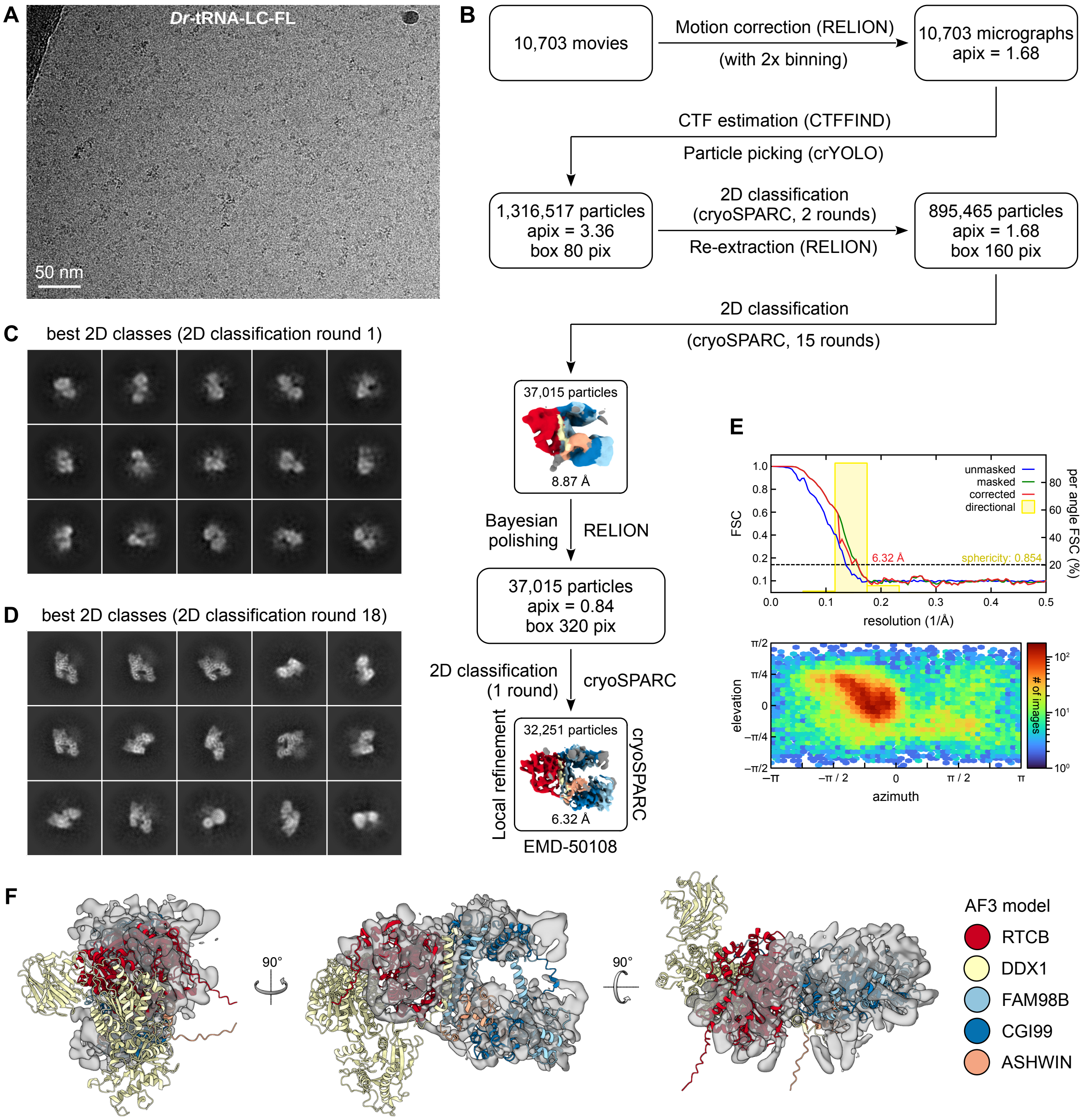

Supplementary Figure 1. ***Dr*-tRNA-LC-FL cryo-EM data processing pipeline**. (**A**) Representative cryo-EM micrograph. (**B**) Initial processing steps and three-dimensional reconstruction pipeline. (**C**) Best (most abundant) 2D classes obtained for all picked particles. (**D**) Best (most abundant) 2D classes obtained after the final 2D classification round. (**E**) Top: gold-standard Fourier Shell Correlation (FSC) curves between two half maps (blue: unmasked, green: masked, red: masked with correction by noise substitution), model-to-map FSC curve (red violet), and histogram of directional FSC (yellow), along with the value of sphericity, calculated by the 3DFSC web-server (61). The horizontal line represents a value of 0.143. Bottom: viewing direction distribution graph. (**F**) AlphaFold 3 model fitted into the final cryo-EM map.

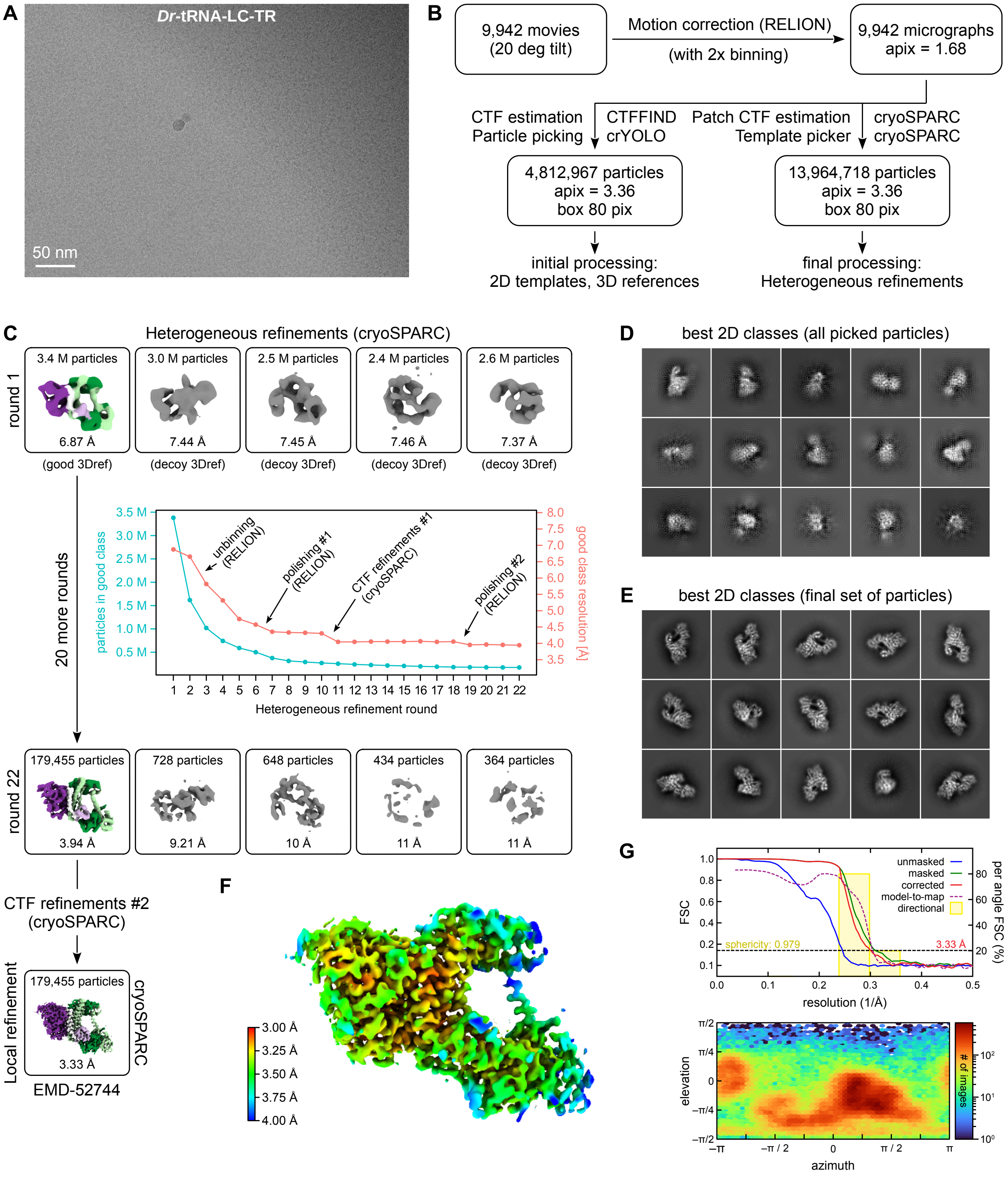

Supplementary Figure 2. ***Dr*-tRNA-LC-TR cryo-EM data processing pipeline**. (**A**) Representative cryo-EM micrograph. (**B**) Initial processing steps (pre-processing, particle picking and extraction). (**C**) Three-dimensional reconstruction pipeline: selection of best particles by iterative heterogeneous refinements and final 3D refinement. 3Dref: three-dimensional references obtained during initial processing. (**D**) Best (most abundant) 2D classes obtained for all picked particles. (**E**) Best (most abundant) 2D classes obtained for the final set of particles. (**F**) Local resolution calculated from half maps in cryoSPARC. (**G**) Top: gold-standard Fourier Shell

Correlation (FSC) curves between two half maps (blue: unmasked, green: masked, red: masked with correction by noise substitution), model-to-map FSC curve (red violet), and histogram of directional FSC (yellow), along with the value of sphericity, calculated by the 3DFSC web-server (61). The horizontal line represents a value of 0.143. Bottom: viewing direction distribution graph.

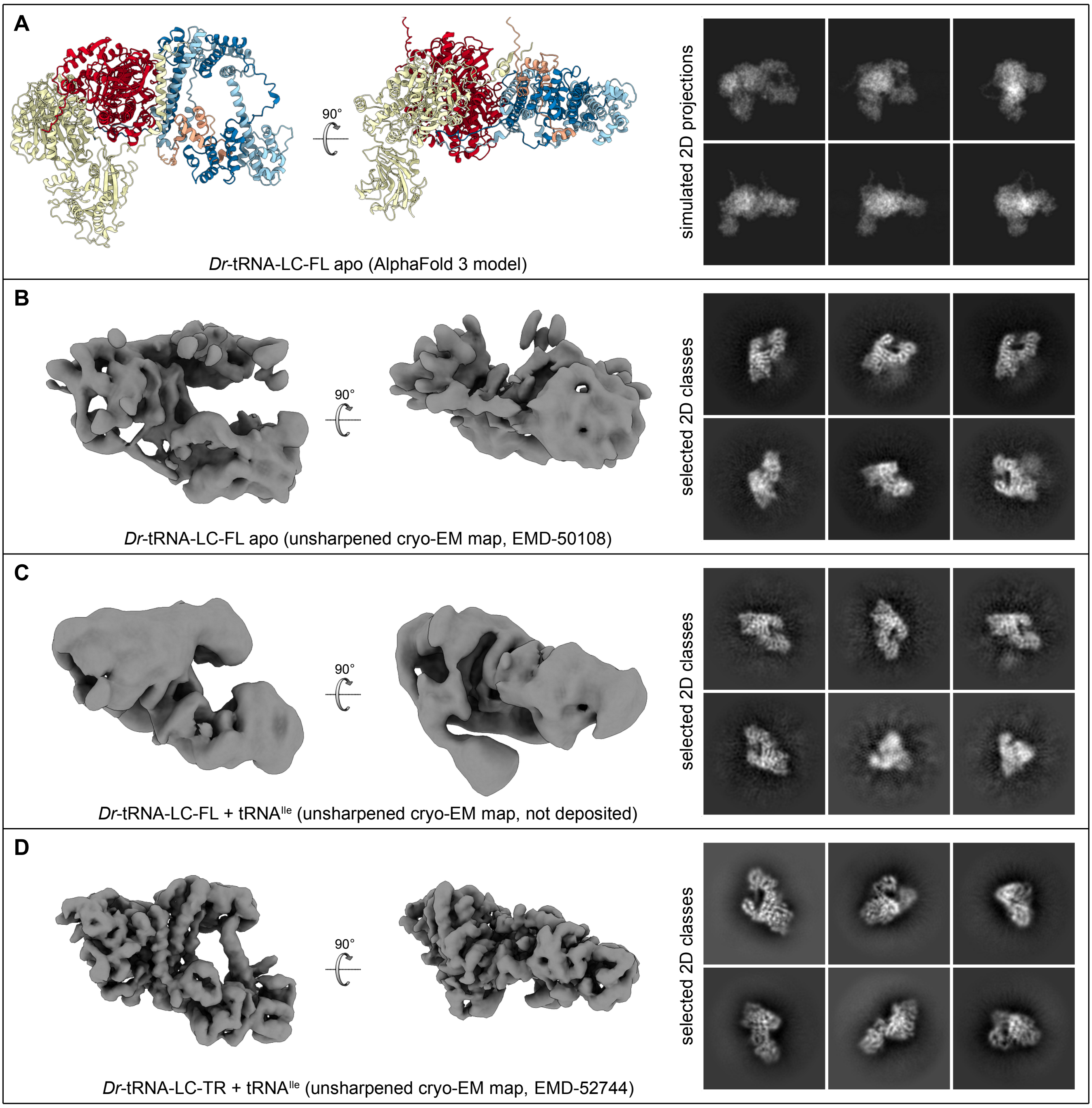

Supplementary Figure 3. **Comparison of *Dr*-tRNA-LC models, maps, and 2D classes.** (**A**) AlphaFold 3 model for *Dr*-tRNA-LC-FL (two views) and simulated 2D projections. (**B**) *Dr*-tRNA-LC-FL unsharpened cryo-EM map (two views) and selected (best) 2D classes. (**C**) *Dr*-tRNA-LC-FL + $tRNA^{Ile}$ unsharpened low resolution cryo-EM map (two views) and selected (best) 2D classes. (**D**) *Dr*-tRNA-LC-TR unsharpened cryo-EM map (two views) and selected (best) 2D classes.

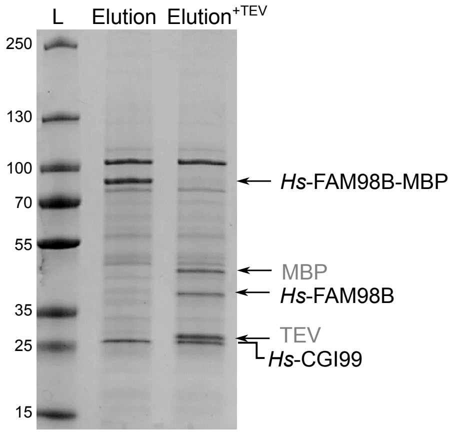

Supplementary Figure 4. **Purification of the human FAM98B–CGI99 heterodimeric complex from insect cells**. Elution – elution fraction from the first HisTrap purification, Elution^{+TEV} – elution fraction digested with TEV protease. L denotes molecular weight marker.

Supplementary Table 1. **Cryo-EM data collection, refinement, and validation statistics**.

	***Dr*-tRNA-LC-FL**<br>**EMD-50108**	***Dr*-tRNA-LC-TR**<br>**EMD-52744**<br>**PDB ID: 9I8V**
**Data collection and processing**		
Microscope	Titan Krios G3i	
Camera	K3 camera (Gatan)	
Energy filter	Gatan BioQuantum with 20 eV slit	
Voltage (kV)	300	
Magnification	105,000×	
Electron exposure (e/Å^2)	39.41	41.09
Defocus range (μm)	-2.1 to -0.9	-2.5 to -1.5
Stage tilt (°)	0	20
Pixel size (Å)	0.86	0.84
Initial particle images (no.)	1,316,517	13,964,718
Final particle images (no.)	32,251	179,455
Symmetry imposed	C1	C1
Map resolution (masked, Å)<br>FSC threshold<br>Map resolution range (Å)	6.32<br>0.143<br>5.8 to 12	3.33<br>0.143<br>2.94 to 8.2
Map sharpening *B*-factor (Å^2)	-457	-168 / Local Filtering
**Refinement**		
Initial models used<br>(source/PDB codes)		ModelAngelo<br>AlphaFold 3
Model composition<br>Non-hydrogen atoms<br>Protein residues<br>Nucleotides<br>Ligands		 7,728<br>1,012<br>0<br>0
Model resolution (Å)<br>FSC threshold		3.5<br>0.5
Model resolution range (Å)[b]		2.95 to 5.05
Model-to-data CC_{volume}		0.79
B-factors (protein, Å^2)		67.85
RMSD<br>Bond lengths (Å)<br>Bond angles (°)		 0.003<br>0.590
Validation<br>MolProbity score<br>Clash score<br>CaBLAM outliers (%)<br>Rotamer outliers (%)		 1.54<br>7.89<br>1.65<br>0.00
Ramachandran plot<br>Outliers (%)<br>Allowed (%)<br>Favored (%)		 0.00<br>2.52<br>97.48

[b] Range of the local resolution map values at atom positions.
